# Image Captioning Using Motion-CNN with Object Detection

**DOI:** 10.3390/s21041270

**Published:** 2021-02-10

**Authors:** Kiyohiko Iwamura, Jun Younes Louhi Kasahara, Alessandro Moro, Atsushi Yamashita, Hajime Asama

**Affiliations:** 1 Department of Precision Engineering, The University of Tokyo, 7-3-1, Hongo, Bunkyo-ku, Tokyo 113-8656, Japan; louhi@robot.t.u-tokyo.ac.jp (J.Y.L.K.); alessandromoro.italy@ritecs.co.jp (A.M.); yamashita@robot.t.u-tokyo.ac.jp (A.Y.); asama@robot.t.u-tokyo.ac.jp (H.A.); 2 RITECS Inc., 3-5-11-403, Shibasakityo, Tatekawa-shi, Tokyo 190-0023, Japan

**Keywords:** deep learning, image captioning, motion estimation, object detection

## Abstract

Automatic image captioning has many important applications, such as the depiction of visual contents for visually impaired people or the indexing of images on the internet. Recently, deep learning-based image captioning models have been researched extensively. For caption generation, they learn the relation between image features and words included in the captions. However, image features might not be relevant for certain words such as verbs. Therefore, our earlier reported method included the use of motion features along with image features for generating captions including verbs. However, all the motion features were used. Since not all motion features contributed positively to the captioning process, unnecessary motion features decreased the captioning accuracy. As described herein, we use experiments with motion features for thorough analysis of the reasons for the decline in accuracy. We propose a novel, end-to-end trainable method for image caption generation that alleviates the decreased accuracy of caption generation. Our proposed model was evaluated using three datasets: MSR-VTT2016-Image, MSCOCO, and several copyright-free images. Results demonstrate that our proposed method improves caption generation performance.

## 1. Introduction

Automatic generation of image captions has been an active computer vision research topic [1]. Various important applications exist, such as indexing services for images on social networks and the depiction of visual contents for visually impaired people. Traditionally, humans generate such captions manually for each image, which is a labor intensive process. Therefore, a need exists for automatic image caption generation.

Recently, deep learning models have been used for the automatic generation of image captions [2,3]. Such models, by training using numerous images, generate accurate image captions for unknown images without the need for humans to design features. Vinyals et al. [2] proposed a model consisting of an image feature extraction component and a caption generation component. This model obtains image features from the input image and then generates captions according to the inputted image features. For image feature extraction, convolutional neural networks (CNN) were used. For captions, recurrent neural networks (RNN) were used. These models can learn relations between overall image features and corresponding words in the caption. Later, other models have been proposed. These models incorporate attention mechanism or object detection to obtain a specific image region, such as in objects. Therefore, the models can learn relations between specific image features and corresponding words in the caption.

Most models used for image captioning improve performance using the overall image or specific image regions. These methods are aimed at direct learning of relations between image features and corresponding image captions. However, this is not relevant for verbs that sometimes have no meaningful relation with image features. Therefore, generating captions that include such verbs is expected to be challenging for those captioning models.

By obtaining motion features from particular image regions, the relation between motion features and verbs can be included in the caption generation process. Therefore, Iwamura et al. [4] proposed a captioning model using motion features. This model uses both image features and motion features estimated from a single image using CNN. They reported improved accuracy when high-quality motion features were used. However, one problem with that method is that motion features obtained by background are used. This can cause the following issues: (1) captions mainly describe contents of objects in the image. Therefore, background features are not of much importance. However, when estimating more features in the background than in the object areas, the captioning model incorporates more background features, which might adversely affect caption generation; (2) the motion estimation accuracy is not always high. Some motion features include errors. Therefore, using all the motion features can engender more opportunities to use the wrong features, which can decrease the caption generation accuracy. The research question in our study is as follows: how to use motion features to improve captioning performance on images?

**Figure 1:**
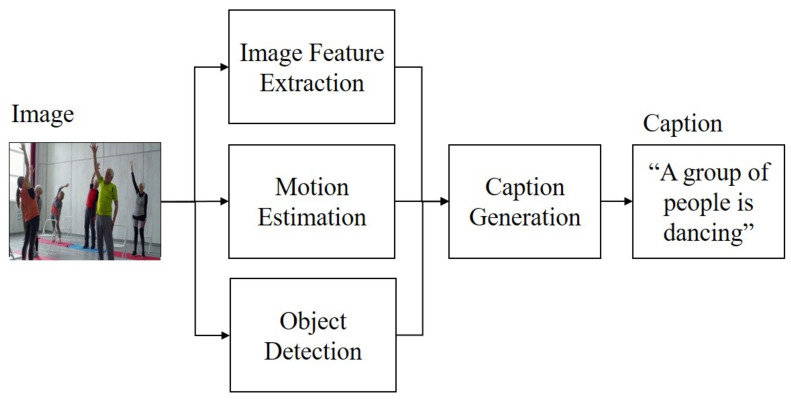
Overview of proposed model. An input image is passed to image feature extraction, motion estimation and object detection components for incorporating image and motion features. Then, the caption generation component is applied for caption generation.

As described herein, we analyze the reasons for the decline in accuracy through experiments with motion features. We also propose a novel method for image caption generation aimed at solving the decrease in the accuracy of caption generation. As presented in Figure 1, the proposed method consists of image feature extraction, motion estimation, object detection and caption generation components.

Overall, the main novelty and contributions of this paper are the following.

- We introduce motion-CNN with object detection. It extracts motion features automatically from object regions in the image.
- We conduct an analysis of our model, particularly of the effects of using only motion features relevant to the object regions, by comparing the error of using all motion features against using only motion features around the object regions.
- We achieve higher accuracy than by earlier methods with MSR-VTT2016-Image and MSCOCO datasets.

## 2. Related Work

A large number of models for image captioning have been proposed. Roughly, these models can be categorized between overall image region models and specific image region models.

Overall image region models use overall image regions to generate captions. Many proposals have been made, and one of the most typical is Vinyals et al. [2]. As mentioned in the Introduction, the model consisted of an image feature extraction component and a caption generation component. This model obtains image features from input image and then generates captions according to the inputted image features using CNN and RNN. A similar model is used thereafter. Wang et al. [5] introduced a captioning model that consisted of an image feature extraction component and a caption generation component. This model had a different caption generation component compared to a captioning model presented earlier in the literature [2]. Particularly, bidirectional long-short term memory (LSTM) for RNN, which performed bidirectional calculations, was used.

Specific image region models adaptively use specific image regions to generate captions. To generate highly accurate captions, it is often important to exploit specific image features, such as objects. Therefore, a visual attention captioning model has been proposed [6]. This model specifically examines the regions of an image related to specific words in image captions by incorporating the attention component into the caption generation component. The attention mechanism here refers to a structure that makes it possible to gaze at specific image features from all image features. For that reason, attention-captioning models have been developed, with high performance reported for image captioning. The first approach using an attention mechanism was that reported by Xu et al. [6]. They improved caption quality, but also provided traceability by providing the ability to visualize what the model used. Lu et al. [7] proposed an adaptive attention mechanism, which decides when to rely on visual features or LSTM memory according to words in the caption. More recently, some studies [1,2,8] proposed an image captioning model with an object detection component. Object detection [9,10] is a method for estimating the position or type of object in an image. Therefore, assisted by object detection, the models explicitly use knowledge about objects in the image. Those models can improve performance related to caption generation. Because the model [1] has image feature extraction, caption generation and object detection components, this model can learn relations between caption words and image features returned by object detection. In other words, those models can generate captions including nouns related to image features obtained by object image regions.

## 3. Method

We explain the concept of the proposed method in Section 3.1. We introduce our proposed captioning models in Section 3.2, Section 3.3 and Section 3.4.

### 3.1. Concept

As discussed in the Introduction, most image captioning models have image feature extraction, object detection, and caption generation components. Those models improve performance using the overall image or specific image regions. However, those features are not relevant for verbs, which have no meaningful relation with image features. For example, if a model is trained to generate the word “walk” from a human with open legs, it means that the word “walk” would also be generated by an image of a human swimming with similarly open legs. This is despite the fact that the correct answer is the word “swim”. Another example is that it is often difficult for a model to generate the word “standing” or “doing gymnastics” from the image of a human who stands indoors and does gymnastics with hands up. Therefore, in such a case, a typical captioning model often cannot generate a verb and generates the caption “human in a room”. Therefore, a model [4] that adopts motion estimation component was proposed. This model can learn the relation between motion features and verbs in cases where it is difficult to learn a relation between image features and verbs. However, as described in the Introduction, motion features obtained in the background are used. This shortcoming can lead to two important difficulties. First, captions mainly describe the contents of objects in the image. Therefore, background features are not of great importance. However, when estimating more features in the background than in the object areas, the captioning model incorporates more background features, which might adversely affect caption generation. Second, the accuracy of the motion estimation is not always high. Some of the motion features include some errors. Therefore, using all of the motion features can engender more opportunities to use the wrong features, which can decrease the accuracy of caption generation. To resolve this difficulty, we use only motion features around the object region of an image. With respect to the former case, the caption model can specifically examine important image regions for image captioning. With respect to the latter case, the opportunities for the caption model to use the wrong features are lessened.

As described in this paper, our concept is to use only motion features obtained in object regions of an image. Based on the points above, our model needs all of image feature extraction, motion estimation, object detection, and caption generation components, which have been overlooked in methods described in earlier reports of the relevant literature. Using image feature extraction, our model can obtain rich image information, such as the shape and color of objects, as in most previous works. The caption generation component is used for various captions according to image features. By incorporating motion estimation, our model can learn meaningful relations between image features and words via motion features, which is what most earlier methods failed to achieve. Using object detection, our model can alleviate the decrease in the accuracy of caption generation caused by simply using all motion features, which is what no model was able to achieve. Furthermore, the proposed model can be incorporated into most of the earlier methods.

### 3.2. Overall Model Architecture

As described in the introduction about Figure 1, the proposed method consists of image feature extraction, motion estimation, object detection and caption generation components. The reason for this is that, as described in the concept, only motion features around object regions are considered for use. In other words, we want to use only the motion features of the object region, not all the motion features. Therefore, object detection that detects an object region in an image is incorporated in our model.

The proposed methods of image feature extraction, caption generation, object detection, and motion estimation components for image feature extraction and caption generation have been described earlier in the literature [1,4,6]. The image feature extraction component is used for incorporating image features. The caption generation component is used for generating captions. In the motion estimation component, as described by Iwamura et al. [4], motion-CNN is incorporated. However, this model simply uses all motion features, which might cause a loss of accuracy. Therefore, we introduce an object detection motion estimation component. Specifically, novel motion-CNN with object detection is proposed.

**Figure 2:**
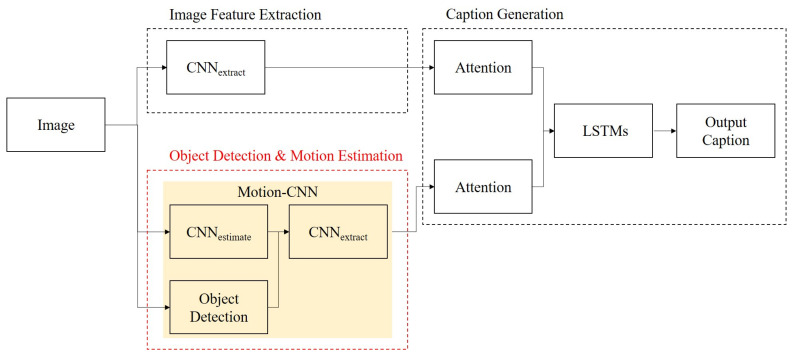
Overview of our proposed model, which comprises ${\mathrm{CNN}}_{\mathrm{extract}}$ and motion-CNN, consisting of ${\mathrm{CNN}}_{\mathrm{estimate}}$, ${\mathrm{CNN}}_{\mathrm{extract}}$, and object detection. Attentions and long-short term memories (LSTMs) are performed to obtain attention features and captions. The model takes the images and the words estimated at the last time step as input.

Figure 2 presents an overview of our model. Figure 2 shows the details of Figure 1, and the elements corresponding to Figure 1 are described in Figure 2, such as image feature extraction and caption generation components. In other words, each component in Figure 1 is described with deep neural networks in Figure 2. An image is passed to the image feature extraction component and the object detection motion estimation component. During image feature extraction, ${\mathrm{CNN}}_{\mathrm{extract}}$ extracts image features. During object detection motion estimation, the image is passed to ${\mathrm{CNN}}_{\mathrm{estimate}}$ and ObjectDetection. Then, ${\mathrm{CNN}}_{\mathrm{estimate}}$ estimates motion features from an input image and ObjectDetection detects objects in the input image. Afterwards, ${\mathrm{CNN}}_{\mathrm{extract}}$ extracts motion features from the input. Next, in the caption generation component, attention features are calculated using Attention. Then, concatenated attention features are inputted to LSTMs. The caption is generated.

### 3.3. Object Detection

Object detection is used for obtaining object regions from an image. The proposed model can ascertain an object region of an image. Therefore, the object detection component is incorporated into our model. As described herein, Faster R-CNN [10] is used for object detection. This model is among the most-used models among object detection methods. By inputting an image, the model outputs bounding boxes. Each box represents a detected object region (location and its size). These bounding boxes are then used as inputs to the ${\mathrm{CNN}}_{\mathrm{extract}}$.

### 3.4. Motion CNN with Object Detection Architecture

As discussed in the concept, to combine object detection into the proposed model, we propose a novel network architecture.

Similarly to Iwamura et al. [4], motion-CNN is incorporated into motion estimation. However, because this model simply uses all motion features, this might lead to a loss of accuracy, as discussed in the concept. Therefore, we propose novel motion-CNN with object detection.

**Figure 3:**
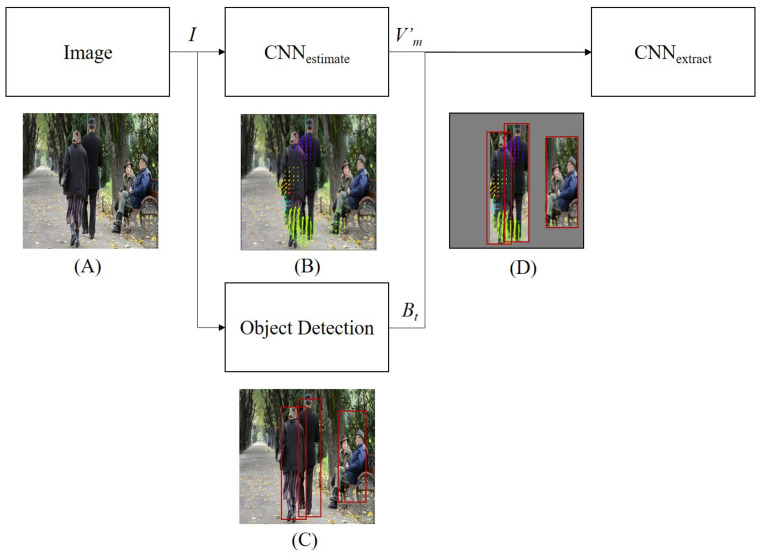
Overall architecture of motion-convolutional neural networks (CNN) and examples corresponding to input image: (**A**) input image example and (**B**) depiction of estimated optical flow. Following Gao et al. [11], we use same-color coding to visualize flow for best viewed: (**C**) depiction of estimated object detection and (**D**) flow with only the object area remaining.

Figure 3 presents an overview of our motion-CNN with an object detection model. It consists of CNNs of two types and object detection. Given an image, convolutional motion estimation is first conducted. The output is image expressed motion of the same size as the input image. This expressed motion is estimated as optical flow. Optical flow is the pattern of apparent motion of objects or edges in a visual scene generated by sequential images. Optical flow has information about motion. However, in the image captioning field, the model’s input is a single image. Therefore, it is impossible to calculate the optical flow. To resolve this difficulty, the estimated optical flow is used in our model. The estimated optical flow is the pattern of apparent motion of objects or edges in a visual scene generated by a neural network. Therefore, a neural network is incorporated to estimate the optical flow in our model. At the same time, object detection is performed. We can acquire regions in the image where objects might be present. Instead of using all the estimated optical flow, we use features only in the region where objects are detected. Specifically, the motion features in the region where the object is kept are retained as original values. The features in other regions are set to 0. Then, convolutional feature extraction is applied and motion features are obtained from images.

The process is definable by the following formulas.

(1) $$V_{m}^{\prime }={\mathrm{CNN}}_{\mathrm{estimate}}\left(I\right),$$

(2) $$B_{t}=\mathrm{object}-\mathrm{Detection}\left(I\right),$$

(3) $$V_{m}^{\prime }=\left\{\begin{array}{cc} V_{m}^{\prime } & \left(i\in B_{t}\right)\\ 0 & (otherwise), \end{array}\right.$$

(4) $$V_{m}={\mathrm{CNN}}_{\mathrm{extract}}\left(V_{m}^{\prime }\right),$$

In those equations, $V_{m}^{\prime }$ stands for the ${\mathrm{CNN}}_{\mathrm{estimate}}$ output (same size as the input image). Moreover, ${\mathrm{CNN}}_{\mathrm{estimate}}\left(\cdot \right)$ denotes the motion estimation. $B_{t}$ are bounding boxes obtained by object detection at time step *t*. object-detection(·) shows object detection. *i* denotes a pixel of $V_{m}^{\prime }$ and ${\mathrm{CNN}}_{\mathrm{extract}}\left(\cdot \right)$ signifies feature extraction. $V_{m}$ represents the ${\mathrm{CNN}}_{\mathrm{extract}}$ output.

As might be apparent from the explanation above, with the proposed motion-CNN as the inputted image, the model can generate motion features related to object regions automatically from an inputted image with no help.

## 4. Experiments

### 4.1. Implementation Details

For our experiments, we used ResNet-101 [12] pretrained on ImageNet [13] for ${\mathrm{CNN}}_{\mathrm{extract}}$ to extract image features. The reasons for choosing ResNet-101 is that ResNet-101 is widespread and boasts the highest performance for image captioning. Im2Flow [11] was used for ${\mathrm{CNN}}_{\mathrm{estimate}}$ to estimate motion features from images. The reason for choosing Im2Flow is that, there is only one network, Im2Flow, which can estimate motion features from a single image. We fine-tuned ${\mathrm{CNN}}_{\mathrm{extract}}$ for the MSR-VTT2016-Image. We used the Adam optimizer with base learning rate of $2\times 10^{-4}$ for the LSTMs and $1\times 10^{-5}$ for the ${\mathrm{CNN}}_{\mathrm{extract}}$ for the MSR-VTT2016-Image. The base learning rate of $1\times 10^{-4}$ was used for the LSTMs for MSCOCO. The decay rate was set to 0.8 for every 3 and 5 epochs for MSCOCO and MSR-VTT2016-Image. The dimensions of embedding layers and both LSTMs were set to 512. For MSCOCO, captions are represented as a GloVe feature [14]. We trained our model under cross-entropy loss with doubly stochastic regularization [6]. For MSCOCO, cross-entropy loss is used. In the decoding process, we used beam search with the beam size set to 3. We set the batch size to 16. All experiments were conducted using a computer system (Ubuntu 18.04; Core i9-7900X CPU, Intel Corp. with 64G RAM memory; GTX2080 Ti GPU with 12G memory). We used PyTorch for a deep learning framework (version 1.0). Following Lu et al. [7], we truncated captions longer than 18 words for MSCOCO and 22 for the MSR-VTT2016-Image. We then built a vocabulary of words, particularly removing words that occurred fewer than five times, and obtained a vocabulary of 9486 and 7802 words for MSCOCO and MSR-VTT2016-Image. Parameters were essentially reused from Anderson et al. [1], Lu et al. [7] and Iwamura et al. [4]. However, for learning rate and decay rate, multiple values are tried and the best ones were used. In our research, the images in the dataset are split into training, validation, and test sets. We used Karpathy split and Xu split to split the dataset, as in Lu et al. [7] and Xu et al. [15] for the MSCOCO and MSR-VTT2016-Image dataset, respectively. It is common in this image captioning field to train a model by dividing it into three parts (training, validation, test). These divided datasets are used to train the model. The source code is available on the website https://github.com/K-Iwamura/MotionCNN-with-ObjectDetection (accessed on 10 February 2021).

Four experiments were conducted.

- Experiment 1 specifically emphasized examination of the accuracy of motion features between object regions and other regions. Specifically, the MSR-VTT2016-Image is used to measure the error between the estimated motion features and the ground truth motion features. Motion features consist of three channels, which are an angle in the x direction, an angle in the y direction, and its magnitude. For calculating error, mean square error (MSE) is used.
- Experiment 2 performed image captioning with copyright-free images that are freely available on the internet. We checked the operation of our model and compared our model with other models.
- Experiment 3 aimed at analyzing the performance of our model using MSR-VTT2016-Image. We compared our model with other models.
- Experiment 4 performed image captioning with MSCOCO. We compared our model with other models.

The following methods were compared in our experiments.

- Previous method [1]: This model included image feature extraction, caption generation, and object detection components. The model is re-implemented as described in the original paper. Except for activation functions, we used the ReLU function. For experiment 3, ResNet-101 [12] was used for image feature extraction. For experiment 4, faster R-CNN [10] with ResNet-101 [12] was used for image feature extraction.
- Previous method [6]: This model included image feature extraction and caption generation components. The model is re-implemented as described in the original paper. Except for activation function, we used the ReLU function. ResNet-101 [12] was used for image feature extraction.
- Previous method [7]: This model included image feature extraction and caption generation components. The model has an adaptive attention mechanism, which decides when to rely on visual features or LSTM’s memory according to the words in the caption. The result is quoted from the original paper.
- Previous method [4]: This model included image feature extraction, caption generation, and motion estimation components. The model is re-implemented as an original paper. The model used simply all the motion features. ResNet-101 [12] was used for image feature extraction.
- Proposed method (with motion estimation): Our proposed model includes motion-CNN image feature extraction, caption generation, motion estimation, and object detection components. For motion features, estimated optical flow was used. These were obtained using neural networks from a single image. (With optical flow): Our proposed model has optical flow for motion features. Optical flow was calculated using two consecutive images that provide high-quality motion features. For experiment 3, ResNet-101 [12] was used for image feature extraction. For experiment 4, faster R-CNN [10] with ResNet-101 [12] was used for image feature extraction.

### 4.2. Detailed Workflow

To explain the whole process, a detailed workflow from data preparation to caption generation is described below. In our research, the images in the dataset are split into training, validation, and test sets. We used Karpathy split and Xu split to split the dataset as in [7,15] for the MSCOCO and MSR-VTT2016-Image datasets, respectively. It is common in this image captioning field to train a model by dividing it into three parts (training, validation, test). These divided datasets are used to train the model. Next, we describe the detailed workflow of inputting the prepared dataset into the proposed model. As we discussed in the Method section, the proposed method has image feature extraction, motion estimation and object detection components. Therefore, input image is passed to these three components at first. Then, in image feature extraction component, ${\mathrm{CNN}}_{\mathrm{extract}}$ extract the image features from input image. At the same time, in motion estimation and object detection components, ${\mathrm{CNN}}_{\mathrm{estimate}}$ estimate motion from input image. Object detection is conducted. Then, obtained results are passed to ${\mathrm{CNN}}_{\mathrm{extract}}$ for extracting motion features. Next, image features and motion features are passed to attention independently. Then, the two features acquired from attention are concatenated and passed on caption generation component. In the caption generation component, LSTMs generate captions according to concatenated features. This provides us with captions. Once the image is inputted, the proposed method is automated, so that there is no need for human involvement.

### 4.3. Datasets

**MSR-VTT2016-Image**. This dataset is an image captioning dataset created using MSR-VTT2016 [15]. Actually, MSR-VTT2016 is a large-scale video dataset with 10,000 clips totaling 41.2 h and 20 captions per clip. To evaluate the effects of motion feature quality, we needed high-quality motion features. Therefore, we used optical flow for this study. Optical flow is the pattern of apparent motion of objects or edges in a visual scene calculated from two consecutive images. For conversion to an image captioning dataset, 4 frames, separated by 10 frames each, were extracted from each clip of MSR-VTT2016 and were associated with five of the corresponding captions. As following Xu et al. [15], we used the same video split. As a result, MSR-VTT2016-Image has 20699, 1460 and 9292 images for training, validation, and test data, respectively. To generate optical flow, we used LiteflowNet2 [16], which produced an image-captioning dataset with the benefit of actual motion associated with each image, i.e., high-quality motion features.

**MSCOCO**. MSCOCO [17] is a large-scale image dataset. This dataset has images associated with five corresponding captions. We use the Karpathy split, which has 113,287, 5000, and 5000 images, respectively, for training, validation, and test data. Therefore, this dataset does not include optical flow.

**Copyright free images**. This dataset, which is used for qualitative analysis, was created using copyright-free web video clips https://pixabay.com/ja/videos/ (accessed on 10 February 2021), in the same fashion as for MSR-VTT2016-Image. Therefore, those images do not include captions.

### 4.4. Evaluation Metrics

To evaluate our model’s performance on image captioning, the commonly used BLEU-N (*N* = 1, 2, 3, 4) metrics [18] were used.

BLEU-N metrics are calculated as

(5) $$BLEU_{N}=\min\left(1,e^{1-\frac{r}{c}}\right)\cdot e^{\frac{1}{N}\sum_{n=1}^{N}\log p_{n}},$$

where *r* represents the reference sentence, *c* denotes the generated sentence, and $p_{n}$ is the modified n−gram precision. We also used METEOR metric [19], ROUGE-L metric [20], and CIDEr metric [21] for comparison. These values fundamentally translate the similarity of the generated caption with the ground truth caption. For all evaluation metrics, higher values represent better results.

### 4.5. Results of Experiment 1

Table 1 presents results obtained from experiment 1. The evaluation metric is MSE, and lower values show better results. MSE between the estimated motion feature and ground truth using the overall image regions was 3743.7. Both features are calculated using each corresponding value (both features have the same overall image regions). MSE between the estimated motion feature and the ground truth using object image regions was 2207.8. Both features are calculated using each corresponding value (both features have the same object image regions). The result was better when only the object image regions were used.

### 4.6. Results of Experiment 2

**Figure 4:**
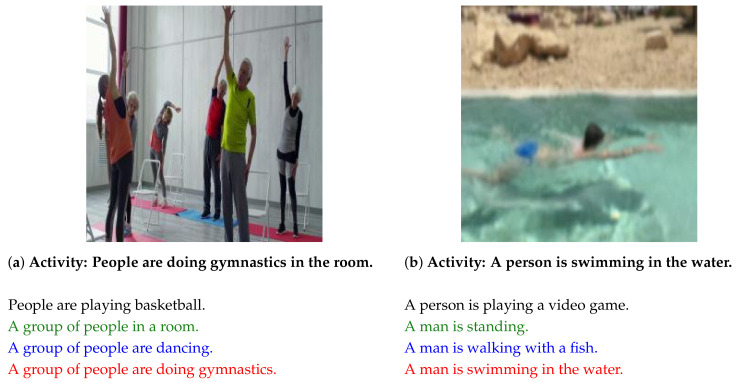
Images used in experiment 2 and captions generated using each method. Activity represents the image content. Activity (**a**) is that people are doing gymnastics in the room. Activity (**b**) is that a person is swimming in the water. Captions in black font were generated using the previous method [6]. Captions in green font were generated using the previous method [1]. Captions in blue font were generated using an earlier method [4]. Captions in red font were generated using our proposed method.

Figure 4 presents copyright-free images used for experiment 2. Figure 4a presents an image of people doing gymnastics. Captions are generated using the respective methods. The earlier method [6] generated “People are playing basketball.” The earlier method [1] generated “A group of people in a room.” An earlier method [4] outputted “A group of people are dancing.” The proposed method generated “A group of people are doing gymnastics.” Using motion features, the earlier method [4] and the proposed method outputs **“dancing”** and **“doing gymnastics”**.

Figure 4b presents an image of a swimming person. Captions are generated using the respective methods. The previous method [6] outputted “A person is playing a video game.” The previous method [1] generated “A man is standing.” An earlier method [4] outputted “There is a man is walking with a fish.” The proposed method generated “A man is swimming in the water.” Compared to earlier methods, the proposed method outputted captions that are more appropriate for the image contents.

**Figure 5:**
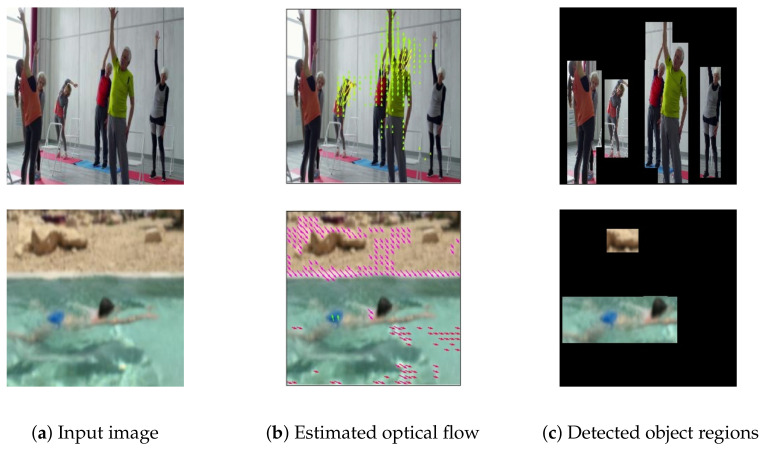
Flow visualization for Figure 5: (**a**) input image, (**b**) optical flow estimated by ${\mathrm{CNN}}_{\mathrm{estimate}}$, and (**c**) detected object regions. Following [11], we use same color coding to visualize flow for most viewed.

Figure 5 presents the flow visualization and the detected object regions of each input image. Similarly to Gao et al. [11], we use the same color coding to visualize the flow for the most viewed.

### 4.7. Results of Experiment 3

Table 2 presents the results obtained from experiment 3. For all evaluation metrics, the best value for each metric is shown using bold typefaces. Higher values represent better results. The proposed method with motion estimation obtained the best results for almost all considered metrics. The proposed method with optical flow was the second best compared to other methods. Results show that our proposed method improved the image captioning performance.

### 4.8. Results of Experiment 4

To evaluate the proposed method using a larger dataset, experiment 4 was conducted. Table 3 presents results obtained from experiment 4. Similarly to Table 2, for all evaluation metrics, the best value for each metric is shown using bold typeface. Higher values represent better results. The proposed method produced the best results for almost all considered metrics. Therefore, our proposed method demonstrably improved the image captioning performance compared with other models.

### 4.9. Discussion

Results obtained from experiment 1 show that MSE is better value only around the object image region, but not when considering the entire image region. These results indicate that using all the motion features can engender more opportunities to use the wrong features.

Results obtained from experiment 2 demonstrate that, as shown in Figure 5, the first row shows that the estimated optical flow corresponds to object regions. These regions are detected correctly by object detection. Therefore, almost identical estimated optical flows are used in the earlier method [4] and the proposed method. However, generated captions differ, which indicates that our ${\mathrm{CNN}}_{\mathrm{extract}}$ can extract motion features more appropriately to generate captions compared to an earlier method [4]. A possible reason for this better generation is that, when training our model, removal of the unnecessary motion features from the background can be regarded as having had a positive effect. The second row shows that the estimated optical flow does not correspond well to object regions. However, these regions are detected correctly by object detection. Therefore, by removing motion features from the background, the proposed method can specifically examine motion features in an object region. Then, the proposed method can generate captions appropriately.

Results obtained from experiment 3 demonstrate that our proposed method improves image captioning performance. Earlier methods [1,6] that do not use motion features have the lowest performance. An earlier method [4] that uses simple overall motion features has better performance, but it is still outperformed by our proposed method. Therefore, the proposed method is effective because it shows the best values in all evaluation metrics. Using object image region motion features has a good effect on our model. Using all motion features is having a negative effect in this experiment 3.

Results obtained from experiment 4 show that our proposed method improves image captioning performance. Previous methods [6,7], that use no motion estimation and object detection components, have the lowest performance. The method of Iwamura et al. [4] that uses simple overall motion features has better performance compared to Xu et al. [6], Lu et al. [7], but it is still outperformed by our proposed method. An earlier method [1] that uses no motion estimation component is only slightly better, being outperformed by our proposed method. Therefore, the proposed method is effective, because it shows the best values in almost all evaluation metrics.

The advantages of our proposed method are most apparent with images containing moving objects: the proposed method achieves its best performance in such cases. This is in particular the case when a moving human is in the image. The reason for this is that our ${\mathrm{CNN}}_{\mathrm{estimate}}$ is pre-trained with human motion. Therefore, relatively high accuracy of estimation of motion features for moving humans, compared to other moving objects, is obtained. In this paper, the specific actions are, for example, “walking with a dog” and “hammer throw”. Those are contained in fast human motion dataset [22] used for pre-training, having 101 action categories. By taking advantage of this motion feature, we can achieve higher performance in image caption generation with verbs.

The potential disadvantages of our proposed method lie in its use of object detection: in the rather rare cases where object detection significantly fails, the performance of our proposed method could be degraded. However, this rarely happens. Incidentally, we have confirmed that even if the object detection results are wrong to some extent, there is no problem. Moreover, even if the object detection cannot detect a moving object at all, the accuracy would be similar to Iwamura et al. [4]. In addition, the accuracy of the generation of motion features is reduced when the dataset used for pre-training and the data for testing are far apart. For example, when ${\mathrm{CNN}}_{\mathrm{estimate}}$ is pre-trained with fast human motion, the accuracy is lower for test data that include very slow moving objects. Therefore, to address such cases, it must be pre-trained on a different dataset that includes slow-moving objects.

## 5. Conclusions

As described herein, we experimented with motion features to analyze the reasons for the decline in accuracy when using all motion features. After doing so, we proposed a novel method for image caption generation aimed at alleviating the decreased accuracy of caption generation. The proposed method can use only motion features around the object regions of an image. Therefore, the proposed model can eliminate unnecessary motion features for generating image captions, which can prevent decreased accuracy of caption generation. We demonstrated that our proposed method using motion-CNN with object detection improved performance on caption generation and that using motion features around the object regions is better than using all the motion features in our experiments.

Our future work will specifically examine how to incorporate motion features better. As described in this paper, object detection is incorporated into our model to use motion features. However, as described in the discussion presented above, object detection sometimes generates bounding boxes that cover all image regions.

## Figures and Tables

**Table 1:** Results of experiment 1 with MSR-VTT2016-Image dataset. The best value for each metric is shown using bold typeface.

Datasets	MSE (Overall Image Regions)	MSE (Object Image Regions)
MSR-VTT2016-Image	3743.7	**2307.8**

**Table 2:** Results of experiment 3 conducted with the MSR-VTT2016-Image dataset (average of five runs). With optical flow: Our proposed model, with optical flow for motion features. Optical flow was calculated using two consecutive images. With motion estimation: Our proposed model. For motion features, the estimated optical flow was used. The best value for each metric is shown using bold typeface.

Method	BLEU-1	BLEU-2	BLEU-3	BLEU-4	METEOR	ROUGE-L	CIDEr
Previous method [6]	49.4	31.5	21.0	14.2	15.8	39.2	31.4
Previous method [1]	48.8	31.5	21.2	14.4	15.8	39.3	32.1
Previous method [4]	49.5	31.8	21.4	14.5	15.8	39.3	32.5
With optical flow	49.3	31.8	21.4	**14.6**	16.0	39.3	**32.7**
With motion estimation	**49.9**	**32.2**	**21.5**	14.5	**16.1**	**39.5**	**32.7**

**Table 3:** Results of experiment 4 with MSCOCO dataset. Proposed method: For motion features, estimated optical flow was used. The best value for each metric is shown using bold typeface.

Method	BLEU-1	BLEU-2	BLEU-3	BLEU-4	METEOR	ROUGE-L	CIDEr
Previous method [6]	70.7	49.2	34.4	24.3	23.9	-	-
Previous method [7]	74.2	58.0	43.9	33.2	26.6	-	108.5
Previous method [1]	**76.3**	**59.9**	45.9	34.9	26.6	**55.8**	109.4
Previous method [4]	75.5	59.5	45.8	35.0	**26.8**	55.7	108.5
Proposed Method	75.9	**59.9**	**46.0**	**35.2**	26.7	**55.8**	**109.9**

## Data Availability

Publicly available datasets were analyzed in this study. This data can be found here: https://pixabay.com/ja/videos/ (accessed on 10 February 2021).
